# Biosensor for Hepatocellular Injury Corresponds to Experimental Scoring of Hepatosplenic Schistosomiasis in Mice

**DOI:** 10.1155/2016/1567254

**Published:** 2016-06-08

**Authors:** Martina Sombetzki, Nicole Koslowski, Sandra Doss, Micha Loebermann, Michael Trauner, Emil C. Reisinger, Martin Sauer

**Affiliations:** ^1^Division of Tropical Medicine and Infectious Diseases, Center of Internal Medicine II, University of Rostock, 18057 Rostock, Germany; ^2^Fraunhofer Institute for Cell Therapy and Immunology, 04103 Leipzig, Germany; ^3^Hans Popper Laboratory of Molecular Hepatology, Division of Gastroenterology and Hepatology, Department of Internal Medicine III, Medical University of Vienna, 1090 Vienna, Austria; ^4^Anesthesiology and Intensive Care Medicine, Medical Faculty of the University of Rostock, 18057 Rostock, Germany

## Abstract

Severe hepatosplenic injury of mansonian schistosomiasis is caused by Th2 mediated granulomatous response against parasite eggs entrapped within the periportal tissue. Subsequent fibrotic scarring and deformation/sclerosing of intrahepatic portal veins lead to portal hypertension, ascites, and oesophageal varices. The murine model of* Schistosoma mansoni *(*S. mansoni*) infection is suitable to establish the severe hepatosplenic injury of disease within a reasonable time scale for the development of novel antifibrotic or anti-infective strategies against* S. mansoni* infection. The drawback of the murine model is that the material prepared for complex analysis of egg burden, granuloma size, hepatic inflammation, and fibrosis is limited due to small amounts of liver tissue and blood samples. The objective of our study was the implementation of a macroscopic scoring system for mice livers to determine infection-related organ alterations of* S. mansoni* infection. In addition, an* in vitro* biosensor system based on the detection of hepatocellular injury in HepG2/C3A cells following incubation with serum of moderately (50* S. mansoni* cercariae) and heavily (100* S. mansoni* cercariae) infected mice affirmed the value of our scoring system. Therefore, our score represents a valuable tool in experimental schistosomiasis to assess severity of hepatosplenic schistosomiasis and reduce animal numbers by saving precious tissue samples.

## 1. Introduction

In the host adult* Schistosoma mansoni (S. mansoni)* worms are found within the mesenteric blood vessels around the sigmoid colon and the rectum, where the oviposition takes places [1]. Schistosomal eggs induce a strong antibody response against soluble egg antigens that corresponds to a type 4 hypersensitivity reaction mediated by T-helper 2 (Th2) cells and alternatively activated macrophages. Via bloodstream the lateral-spined eggs get into the liver and become entrapped within the periportal tissue [2]. Th2 response directs periocular inflammation towards fibrous scarring with subsequent deformation and sclerosing of the intrahepatic portal veins whereby the arterial and ductular structures remain largely unaffected [3]. Fibrotic septa sprout from the periportal area into the parenchyma, while the acinar architecture is preserved. Due to high infection rates or repeated infections periportal (Symmers' pipestem) fibrosis [4] occurs as mild chronic granulomatous hepatitis or advanced hepatosplenic manifestations with massive fibrosis, splenomegaly, portal hypertension, ascites, and esophageal varices.

By virtue of intact liver function serum biochemistry is inconspicuous. During the acute stage of disease (Katayama syndrome) eosinophilia and a slight increase of alanine aminotransferase (ALT) can be seen. Later in the chronic stage of disease aminotransferase levels are often within the normal ranges and alkaline phosphatase might be slightly elevated.

Apart from discrepancies regarding susceptibility for infection between mice and man [5], immunological reactions to parasite eggs run similarly in terms of involved cells types and regulating cytokines, granuloma formation, disease progression, and clinical symptoms [6]. In man, disease progression towards advanced hepatosplenic schistosomiasis depends on the age, gender, infection rates, and duration of infection [7]. The mouse model is suitable to establish severe hepatosplenic manifestations within eight weeks by infection with low numbers of worms [3]. Therefore the murine model is used to study the complex immune reactions to egg and worm antigens within a reasonable time scale and to develop novel antifibrotic or anti-infective strategies against* Schistosoma* infections [8]. However, complex analysis of egg burden, granuloma size, hepatic inflammation, and fibrosis is limited due to small amounts of liver tissues and blood samples. Therefore usually large numbers of animals are needed, in order to address scientific issues adequately.

The objective of our study was the implementation of a macroscopic scoring system determining infection-related organ alterations as markers of disease severity in mice infected with* S. mansoni*. An* in vitro* biosensor system detecting hepatocellular injury in HepG2/C3A cells affirmed our scoring system.

## 2. Material and Methods

### 2.1. *Schistosoma mansoni* Mouse Model

*Schistosoma mansoni* (*S. mansoni*, Brazilian strain) was held in a life cycle with* Biomphalaria glabrata* fresh water snails (*B. glabrata*, Brazilian strain) as intermediate hosts and female NMRI mice as definite hosts. Mice were kept with a 12:12 hour light/dark cycle, standard mouse chow (SSNIFF, Soest, Netherlands), and water* ad libitum*.* B. glabrata* were kept in aquarium water at 25°C and a lettuce diet. Cercariae were obtained by mass shedding after light exposure and the number of cercariae/mL was determined using a conventional light microscope (magnification 100-fold). To generate pathologies of different severities, 6- to 8-week-old NMRI mice were infected with either 50* S. mansoni* cercariae (*n* = 9) for a moderate infection or 100* S. mansoni* cercariae (*n* = 9) for a heavy infection. Mice were exposed to* S. mansoni* cercariae by sitting in a water bath, except the healthy control-group (*n* = 5). Twelve weeks after infection mice were sacrificed via cervical dislocation under Ketamine/Xylazine anesthesia. Collection of sterile plasma for biosensor testing was performed by immediate retrobulbar exsanguination using lithium/heparin blood collection tubes (Sarstedt, Hannover, Germany). Within the experimental groups plasma samples were pooled and frozen at −80°C. The experiments were performed according to the German animal protection law and approved by the local animal care and use committee (file number 7221.3-2.5-003/10).

### 2.2. Serum Biochemistry and Liver Histology

Alanine aminotransferase (ALT), aspartate aminotransferase (AST), alkaline phosphatase (AP), and bilirubin were measured in serum with the UniCel®* DxC 800* Synchron® Clinical System (Beckman Coulter GmbH).

One-half of the right liver lobe was fixed in 10% neutral buffered formalin solution (Sigma Aldrich, Germany) and embedded in paraffin. Five *μ*m thin sections were stained with either hematoxylin/eosin (HE) or Sirius red (SR).

### 2.3. Macroscopic Evaluation of Liver Injury

Twelve weeks after infection, liver and spleen weights were determined and the extent of liver damage was assessed macroscopically by using a minus (−)/plus (+) scoring system: (−) unobtrusive for the evaluated parameter and (+) obtrusive for the evaluated parameter. The score ranges from 1− = healthy to 12+ = heavily affected by* S. mansoni* infection. The scoring system determines the extent of liver damage and considers infection-related organ alterations as color, stiffness, and prevalence of nodules compared to naive livers (Table 1).

### 2.4. Human Hepatocyte Based Biosensor

Two mL heparinized plasma was drawn from all three experimental groups for testing with the* in vitro* hepatotoxicity test [9, 10]. To determine the toxicity of animal plasma the human hepatocytes cell line HepG2/C3A obtained from the American Type Culture Collection (ATCC CRL-10741) was used. The cells were cultivated in Dulbecco's modified Eagle's Medium (Dulbecco's MEM, GIBCO Life Technologies, Eggenstein, Germany). HepG2/C3A cells were seeded in 24-well cell culture plates in a density of 250.000 cells/well; then the cells were cultured for three days with 0.5 mL heparinized plasma from each animal. Subsequently, cells were rinsed once with Dulbecco's MEM and incubated with 1 mL fresh Dulbecco's MEM for three days. Cells, respectively, supernatants, were tested for viability using the XTT test (dehydrogenase activity in the mitochondria) and measurement of cell count was indicated with the trypan blue-staining. The XTT test was carried out according to the protocol of Scudiero et al. [11]. Additionally, the release of lactate dehydrogenase (LDH) into the cell culture supernatants was measured. Each test batch with plasma from one group was tested in four experiments (*n* = 4), Dulbecco's MEM control was used and each measurement was performed twice.

### 2.5. Statistical Analysis

Significance between the groups was analyzed with the Kruskal-Wallis one way and the two-tailed Mann-Whitney *U*-test, using the Statistical Package for the Social Sciences (SPSS). The results are expressed as median and range. Differences were considered significant at *p* ≤ 0.05.

## 3. Results

### 3.1. Serum Biochemistry

**Figure 1:**
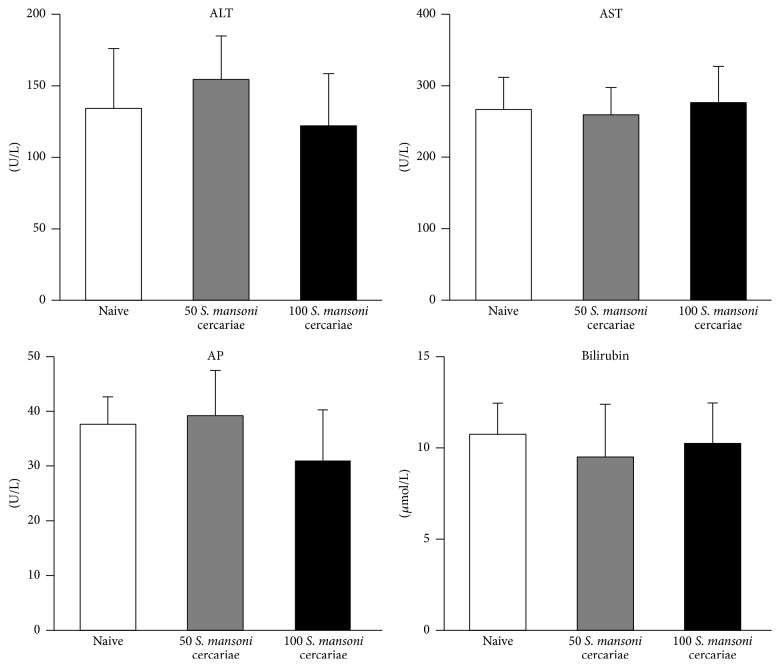
Serum biochemistry of mice infected with 50 or 100* S. mansoni* cercariae. Serum biochemical markers of hepatocellular injury (ALT and AST) and cholestasis (AP and bilirubin) remain unchanged in mice infected with 50 or 100* S. mansoni* cercariae compared to naive control mice.

Serum levels of ALT, AST, AP, and bilirubin were not increased by the different* S. mansoni* infection intensities compared to the naive control (Figure 1).

### 3.2. Scoring System

**Figure 2:**
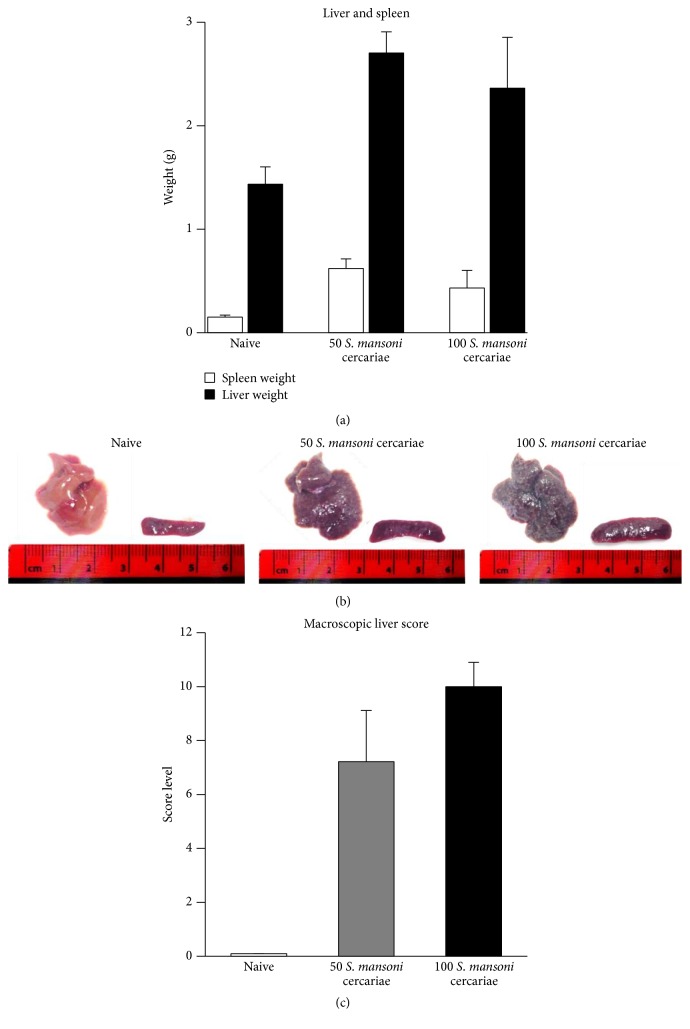
Pathological changes of the liver due to different* S. mansoni* infection intensities. (a) Weights of spleens and livers of infected mice groups are significantly increased compared to naive mice. Infection with 50 or 100 cercariae results in a comparable increase of spleen and liver weights. (b) The outer appearance of mice livers following infection with 50 or 100 cercariae is markedly different. Compared to the livers of naive and mice infected with 50 cercariae, the livers of mice infected with 100 cercariae appear firmer and paler with area-wide nodules, bosselations, and star-like depressions. (c) Scoring of parameters describing the outer appearance of all mice livers reveals highest score levels in mice infected with 100 cercariae compared to livers of naive mice and mice infected with 50 cercariae.

*S. mansoni* infection resulted in significant increase of liver and spleen weights compared to healthy control mice (Figure 2(a)). The different infection intensities were reflected by marked differences in the macroscopic appearance of infected mice livers in mice infected with 100* S. mansoni* cercariae (Figure 2(b)). Livers of these mice appeared greyish and pale; bosselations of the capsule with star-like depression and wide spread nodules were clearly visible. A semiquantitative evaluation of the infection-related organ alteration was performed according to the scoring system described in Table 1. The highest score levels with a mean of 10 ± 0.1 were found in mice infected with 100* S. mansoni* cercariae compared to mice infected with 50* S. mansoni* cercariae and score levels of 7 ± 1.9 (Figure 2(c)).

### 3.3. Microscopic Appearance

**Figure 3:**
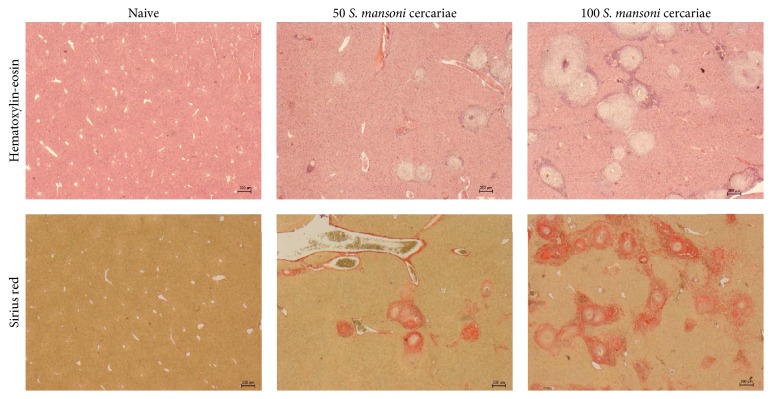
Liver histology of mice infected with 50 or 100 cercariae. Representative images of mice livers (HE, 2.5-fold magnification and SR, 2.5-fold magnification): uninfected mice (naive), mice infected with 50 cercariae (50 cercariae), and mice infected with 100 cercariae (100 cercariae). Livers of mice infected with 100 cercariae have considerably more periocular granulomas compared to the other mice. Higher infection rates result in tortuous vascularization of the portal area and pronounced portoportal bridging with markedly sclerosed hepatic veins.

Histological survey staining (HE, magnification 2.5-fold) of mice livers revealed clearly visible hepatic granulomas within enlarged portal spaces. Livers of mice infected with 100* S. mansoni* cercariae displayed tortuous vascularization of the portal area with replaced blood vessels. Infiltration with inflammatory cells as macrophages/monocytes, granulocytes, and T-lymphocytes was not restricted to periocular regions but to blood vessels and was sporadically found within the parenchymal interface. The amount of hepatic granulomas was conspicuous higher following infection with 100 compared to 50* S. mansoni* cercariae. Sirius red positive area was mostly distributed in livers of mice infected with 100* S. mansoni* cercariae. Livers of these mice displayed pronounced portoportal bridging and markedly sclerosed hepatic veins. In animals infected with 50* S. mansoni* cercariae connective tissue was restricted to periocular areas with dense connective tissue fibers spread into the parenchymal interface (Figure 3).

### 3.4. Biosensor of Hepatocellular Injury

The results of testing the toxicity of the animal plasma from the three experimental groups using human hepatocytes are displayed in Table 2. A markedly impairment of viability was only seen in the infected groups; however, the impairment of viability was more pronounced in the 100 cercariae group compared with the 50 cercariae group. The plasma of the animals from the control-group led to a slightly decrease of the number of cells and extinction in the XTT test and higher values of LDH in cell culture supernatants compared with the medium-control.

## 4. Discussion

Murine models of* S. mansoni* infection were used to imitate human disease patterns and remain indispensable for antischistosomal drug development. The presented experimental scoring system specifically pays attention to infection-related organ alterations resembling severity of disease. It comprises the evaluation of liver characteristics, which were commonly used for the assessment of hepatic fibrosis and related morbidity in humans such as liver stiffness, presence of nodules, and surface condition [12–14]. The different pathologies and therefore the usability of the score were proven by an* in vitro* biosensor system based on the detection of hepatocellular injury in HepG2/C3A cells.

Infection with fifty to hundred* S. mansoni* cercariae represents the usual amount of cercariae used for experimental schistosomiasis [3, 8, 15]. The presented score describes specific organ alterations following infection with either 50 or 100* S. mansoni* cercariae, resembling moderate and heavy infections under experimental conditions. Both infection intensities are incomparable to human infection, since the heaviest infection found in man at autopsy rarely exceeded five worm-pairs per kilogram of body weight [16]. Experimental schistosomiasis is performed with considerably higher numbers of cercariae to provoke severe clinical patterns within a reasonable time scale.

Diagnostic methods in human as transient elastography (FibroScan) or magnetic resonance elastography are used to analyze liver stiffness, occurrence of nodules, or surface conditions to grade hepatic fibrosis and to assess morbidity. In mice these clinical changes have not attracted scientific interest so far. Common approaches to quantify hepatic fibrosis in mice are based on determination of hepatic hydroxyproline content, histological collagen staining, or measurement of profibrotic gene expression [8, 17]. Examining egg secretion in feces or detection of soluble egg antigen in serum seldom reflects real infection burden or organ alterations [18]. Moreover, determination of whole egg or worm numbers in mice livers requires processing of the whole liver but does not reflect the extent of liver damage.

To distinguish between moderate (50 cercariae) and heavy (100 cercariae)* S. mansoni* infection we performed a cell based analysis of hepatotoxicity in the HepG2/C3A hepatocyte cell line [9, 10]. The incubation with plasma of the three experimental groups (naive, 50 cercariae, and 100 cercariae) led to a significant impairment of cell viability in the infected groups only. According to the histological score, the impairment of test cells was more pronounced in the mice infected with 100* S. mansoni* cercariae than in the mice infected with 50* S. mansoni* cercariae. This might result from different levels of chemokines and cytokines in the plasma of the infected animals. In chronic schistosomiasis the degree of hepatic fibrosis correlates with serum levels of interleukin 13 and interleukin 4 [19, 20]. It is debatable to which extent proinflammatory cytokines are involved in the impairment of cell functions and viability in HepG2/C3A cells, since in chronic schistosomiasis Th1 response is suppressed by Th2 cytokines [21]. However, proinflammatory cytokines are known to affect cell viability by causing dysfunction of mitochondria [22], downregulating albumin synthesis [23], and diminishing function of P450 cytochromes like cytochrome P450-1A2, cytochrome P450-2E1, and cytochrome P450-7A1 [24, 25].

So far, this test system was tested in a prospective clinical study addressing hepatotoxicity of plasma from septic and nonseptic patients [9]. We found that the plasma of patients with septic shock impaired cellular functions and viability of HepG2/C3A cells. These values of biosensor-parameters were increased only in survivors compared to nonsurvivors in this study. Another clinical study in patients with severe sepsis or septic shock tested the influence of an extracorporeal granulocyte treatment on the biosensor cells. During the extracorporeal treatments a significant increase of vitality and function of the test cells was seen. These results suggest a positive impact of the extracorporeal granulocyte treatment on the liver cell vitality and function measured in this indirect cytotoxicity test. Against this background and regarding the fact that in schistosomiasis analysis of standard biochemical markers fails to assess severity of disease, it would be prudent to test the HepG2/C3A biosensor in human schistosomiasis.

In conclusion, the presented experimental scoring system displays a useful tool to assess infection-related alterations of the liver indicating severity of hepatosplenic schistosomiasis. Therefore, the score may help to determine effects of therapeutic interventions aiming at hepatic fibrosis or reduction of infection loads. In addition, improved exploitation of scientific information saves precious tissue samples and may reduce numbers of animals for experiments.

## Figures and Tables

**Table 1:** Description of the macroscopic score.

Parameter	Modality	Score level
Spleen weight	<0,1 g (healthy)	−
	>0,11 g	+
	>0,4 g	++

Liver weight	<1,2 g (healthy)	−
	>1,21 g	+
	>2 g	++

Color	Red/glossy	−
	Dark red/fade	+
	Greyish/pale	++

Nodules	None	−
	Occasional	+
	Area-wide	++

External surface	Regular	−
	Furrows/bosselation	+
	Macronodular	++

Consistency	Soft/elastic	−
	Firm	+
	Rigid	++

**Table 2:** Biosensor results (median and range).

	Naive (*n* = 4)	50 cercariae (*n* = 4)	100 cercariae (*n* = 4)	Medium-control (*n* = 4)
*Number of cells* (×1.000)	*833* (825–840)	*610* (575–645)	*560* (545–575)	*1111* (535–1.355)
*Vitality* (%)	*95.2* (93.3–97.1)	*82.8* (80.6–84.9)	*73.8* (64.4–83.2)	*94.9* (91.1–97.3)
*XTT* (extinction/well)	*1.48* (1.25–2.37)	*0.92* (0.84–1.00)^*∗*^	*0.84* (0.51–0.90)^*∗*^	*2.13* (1.72–3.27)^*∗*^
*Release of LDH* (U/L)	*135* (134–136)	*145* (139–150)^*∗*^	*157* (152–161)^*∗*^	*113* (110–123)

^*∗*^ *p* ≤ 0.05 versus naive; LDH: lactate dehydrogenase.

## Abbreviations

ALT: Alanine aminotransferase
AP: Alkaline phosphatase
AST: Aspartate aminotransferase
*B. glabrata*: * Biomphalaria glabrata*
Th1: T-helper cell response type 1
Th2: T-helper cell response type 2
XTT: 2,3-Bis-(2-methoxy-4-nitro-5-sulfophenyl)-2H-tetrazolium-5-carboxanilide
LDH: Lactate dehydrogenase.

## References

[1] Andrade Z. A. (2009). Schistosomiasis and liver fibrosis. *Parasite Immunology*.

[2] Gryseels B., Polman K., Clerinx J., Kestens L. (2006). Human schistosomiasis. *The Lancet*.

[3] Loebermann M., Sombetzki M., Langner C. (2009). Imbalance of pro- and antifibrogenic genes and bile duct injury in murine *Schistosoma mansoni* infection-induced liver fibrosis. *Tropical Medicine & International Health*.

[4] Symmers W. St. C. (1904). Note on a new form of liver cirrhosis due to the presence of the ova of Bilharzia hæmatobia. *The Journal of Pathology and Bacteriology*.

[5] Wilson R. A., Li X., Castro-Borges W. (2016). Do schistosome vaccine trials in mice have an intrinsic flaw that generates spurious protection data?. *Parasites & Vectors*.

[6] Brunet L. R., Dunne D. W., Pearce E. J. (1998). Cytokine interaction and immune responses during *Schistosoma mansoni* infection. *Parasitology Today*.

[7] Vereecken K., Naus C. W. A., Polman K. (2007). Associations between specific antibody responses and resistance to reinfection in a Senegalese population recently exposed to *Schistosoma mansoni*. *Tropical Medicine & International Health*.

[8] Sombetzki M., Fuchs C. D., Fickert P. (2015). 24-*nor*-ursodeoxycholic acid ameliorates inflammatory response and liver fibrosis in a murine model of hepatic schistosomiasis. *Journal of Hepatology*.

[9] Sauer M. The use of human hepatocytes for determining liver function and liver regeneration.

[10] Sauer M., Haubner C., Mencke T. (2012). Impaired cell functions of hepatocytes incubated with plasma of septic patients. *Inflammation Research*.

[11] Scudiero D. A., Shoemaker R. H., Paull K. D. (1988). Evaluation of a soluble tetrazolium/formazan assay for cell growth and drug sensitivity in culture using human and other tumor cell lines. *Cancer Research*.

[12] Kloetzel K. (1962). Splenomegaly in schistosomiasis mansoni. *The American Journal of Tropical Medicine And Hygiene*.

[13] Gerspacher-Lara R., Pinto-Silva R. A., Serufo J. C., Rayes A. A. M., Drummond S. C., Lambertucci J. R. (1998). Splenic Palpation for the Evaluation of Morbidity due to Schistosomiasis Mansoni. *Memorias do Instituto Oswaldo Cruz*.

[14] Lambertucci J. R. (2014). Revisiting the concept of hepatosplenic schistosomiasis and its challenges using traditional and new tools. *Revista da Sociedade Brasileira de Medicina Tropical*.

[15] Cheever A. W. (1969). Quantitative comparison of the intensity of *Schistosoma mansoni* infections in man and experimental animals. *Transactions of the Royal Society of Tropical Medicine and Hygiene*.

[16] Warren K. S. (1966). The pathogenesis of ‘clay-pipe stem cirrhosis’ in mice with chronic schistosomiasis mansoni, with a note on the longevity of the schistosomes. *The American Journal of Pathology*.

[17] Fickert P., Wagner M., Marschall H.-U. (2006). 24-norUrsodeoxycholic acid is superior to ursodeoxycholic acid in the treatment of sclerosing cholangitis in Mdr2 (Abcb4) knockout mice. *Gastroenterology*.

[18] Abdul-Ghani R. A., Hassan A. A. (2010). Murine schistosomiasis as a model for human schistosomiasis mansoni: similarities and discrepancies. *Parasitology Research*.

[19] Schwartz C., Oeser K., Da Costa C. P., Layland L. E., Voehringer D. (2014). T cell-derived IL-4/IL-13 protects mice against fatal *Schistosoma mansoni* infection independently of basophils. *Journal of Immunology*.

[20] Mentink-Kane M. M., Cheever A. W., Wilson M. S. (2011). Accelerated and progressive and lethal liver fibrosis in mice that lack interleukin (IL)-10, IL-12p40, and IL-13R*α*2. *Gastroenterology*.

[21] Herbert D. R., Hölscher C., Mohrs M. (2004). Alternative macrophage activation is essential for survival during schistosomiasis and downmodulates T helper 1 responses and immunopathology. *Immunity*.

[22] Regueira T., Lepper P. M., Brandt S. (2009). Hypoxia inducible factor-1*α* induction by tumour necrosis factor-*α*, but not by toll-like receptor agonists, modulates cellular respiration in cultured human hepatocytes. *Liver International*.

[23] El-Saadany M. A., Rawel H. M., Raila J., El-Dashloty M. S., Schweigert F. J. (2008). Antioxidants modulate the IL-6 induced inhibition of negative acute-phase protein secretion in HepG2 cells. *Cell Biochemistry and Function*.

[24] Nakai K., Tanaka H., Hanada K. (2008). Decreased expression of cytochromes P450 1A2, 2E1, and 3A4 and drug transporters Na^+^-taurocholate-cotransporting polypeptide, organic cation transporter 1, and organic anion-transporting peptide-C correlates with the progression of liver fibrosis in chronic hepatitis C patients. *Drug Metabolism & Disposition*.

[25] Li T., Jahan A., Chiang J. Y. L. (2006). Bile acids and cytokines inhibit the human cholesterol 7*α*-hydroxylase gene via the JNK/c-Jun pathway in human liver cells. *Hepatology*.

